# Are We Doing Enough? Drug-Related Deaths as a Pressing Social Issue

**DOI:** 10.1177/14550725251396864

**Published:** 2025-11-14

**Authors:** Karoliina Karjalainen, Hakkarainen Pekka, Rönkä Sanna

**Affiliations:** 1 3837 Finnish Institute for Health and Welfare, Helsinki, Finland

High-risk drug use can lead to a range of adverse outcomes, with premature death representing one the most severe ones (Bahji et al., 2020). These deaths cause a high number of years of life lost as the deceased are often young people (Onyeka et al., 2015). In addition to major economic and public health burdens, drug-related deaths are surrounded by vast human suffering by the people using drugs, as well as their close ones. Preventing avoidable losses of life requires not only effective methods and interventions, but also the willingness and courage to implement these measures.

Setting aside data comparability issues due to the variations in national reporting practices (EUDA, 2025b; Waal & Gossop, 2013), the number of drug-induced deaths has been high and increasing in many Northern European countries, such as in the Nordic countries (EUDA, 2025a), as well as in the UK (ONS, 2025). While the average rate of drug-related deaths in European Union was 18 per million inhabitants in 2023, this rate was substantially higher in Sweden (64 per million), Norway (63 per million) and Finland (79 per million) (EMCDDA, 2023). Scotland exceeds even these figures with its exceptionally high rate (224 per million) of drug deaths (NRS, 2024) compared to the rest of Europe.

In Finland, the drug situation has shown a long-term deterioration, as evidenced by several indicators (Rönkä & Markkula, 2020). Drug-induced mortality has approximately tripled since the early 2000s, and the number of deaths has reached record-high levels in recent years (Statistics Finland, 2024). Particularly concerning has been the sharp increase in drug-related mortality of young Finns under 25 years of age during the latter half of the 2010s (Statistics Finland, 2024). Finland currently has one of the highest youth mortality rates due to illicit drug use in Europe (EUDA, 2025a).

Despite a rather substantial research literature on drug-related mortality, it remains an insufficiently understood social, public health as well as human rights issue. This thematic section, *Mortality and premature deaths related to drug use and addiction,* aims to shed further light on the multifaceted nature of drug-related deaths. The section has been prepared within the ongoing research project Young Despair (2022–2028), funded by the Strategic Research Council of Finland (Grant number 352600). This multidisciplinary project seeks to provide solutions to break the pathways leading to violent, suicidal and drug-induced deaths of young people (https://outofdespair.fi).

The articles included in this section examine the phenomenon from diverse perspectives:

- the contextual factors surrounding drug-related deaths among young people;
- mortality risks among individuals engaged with health and social services; and
- the experiences of bereaved close ones, with particular attention to grief among people who use drugs and siblings, and the availability – or absence – of support provided to them.

The existing literature on drug-related mortality has focused on individual risk factors of drug-induced deaths. In their article in this thematic section, O’Gorman et al. (2025) examine in-depth health, social and criminal justice records of young people died of drug overdoses in a region of Scotland. Their analysis shows how the fragmented and complex system of care has not been able to respond to their needs starting from behavioural issues and maltreatment in childhood to substance use and mental health issues later in life.

In the articles by Pitkänen et al. (2025) and Scarpa et al. (2025), register data are used to examine mortality risk among treatment-seeking populations. The findings of both of these studies show that substance-related causes as well as suicides are more common than other causes of death within these populations. Pitkänen et al. (2025) also found that the risk of death remains elevated for several years following treatment for substance use disorder. This highlights the importance of long-term follow-up, adequately sustained aftercare and easy re-entry to treatment.

Scarpa et al. (2025) examined the link between education and deaths of despair (alcohol-related mortality, drug-related mortality, suicides) and found that despair-related mortality risk is higher in highly educated individuals than among those with lower educational attainment. This finding was unexpected because it contrasts with evidence from the general population, where higher education is typically a protective factor. Scarpa et al. (2025) call for further research on how educational and socioeconomic factors influence treatment pathways for individuals with substance use disorders because the results suggest that those with higher education may form a particularly vulnerable subgroup unable to fully benefit from their educational resources in managing their substance use problems.

Two articles examine drug-related deaths and the grief they cause from the perspectives of two groups that have been overlooked. Selseng et al. (2025) used interviews to determine the needs for help and support among the people with heavy drug use who have lost their beloved friends due to drug use. In their review article, Jokipii & Aho (2025), on the other hand, focus on the siblings of people who have died from drugs and their need for help. Both groups’ access to help and support is heavily overshadowed by the stigma associated with drug use and the shame it brings.

As shown by the articles published in this thematic section, as well, drug-related deaths are a complex and multifaceted issue with wide-ranging societal impacts. Their prevention requires coordinated and research-informed responses at multiple levels. These include, for example, ensuring timely access to and adequate resourcing of harm reduction and treatment services, promoting early interventions and reducing stigma. Moreover, although there is scientific evidence on effective tools to prevent these deaths, they may remain unimplemented.

In Finland, the alarming trend of drug-induced deaths outlined above has prompted various actors to propose measures aimed at reducing drug-related deaths. During 2021–2023, an expert group compiled 12 recommendations, including opioid substitution treatment, needle and syringe exchange, supervised drug consumption facilities, and the distribution of take-home naloxone, among others (Kailanto & Viskari, 2023). In parallel, the Safety Investigation Authority of Finland (SIA) conducted a themed investigation into drug-related deaths among individuals under 25 years, resulting in eight additional recommendations. In these recommendations, the overarching priority is to build a nationally cohesive service package and service pathway to ensure timely and effective support for individuals at risk (SIA, 2024).

In response to growing public concern, Finnish government has allocated an appropriation of €10m to well-being service counties and non-governmental organizations for the prevention of drug-related deaths among young people (MSAH, 2025). Resources have also been allocated for the treatment of hepatitis C. However, there has been no desire to undertake an evaluation or reform of drug policy. Just recently, for example, the Parliament rejected the citizens’ initiative to open drug consumption rooms in some cities. Furthermore, the government has made substantial cuts to social assistance for disadvantaged people and families. Thus, although some actions have been taken, there is still plenty of tools that could and should be utilized to prevent drug-related deaths.

Reducing drug-related mortality is not only an economic or public health issue, but also a matter of human rights, and thus a moral imperative. We can save lives, alleviate suffering and promote equity by investing in the prevention of drug-related deaths.
